# Safe Robot Trajectory Control Using Probabilistic Movement Primitives and Control Barrier Functions

**DOI:** 10.3389/frobt.2022.772228

**Published:** 2022-03-16

**Authors:** Mohammadreza Davoodi, Asif Iqbal, Joseph M. Cloud, William J. Beksi, Nicholas R. Gans

**Affiliations:** ^1^ The University of Texas at Arlington Research Institute, Fort Worth, TX, United States; ^2^ Department of Computer Science and Engineering, University of Texas at Arlington, Arlington, TX, United States

**Keywords:** motion control, movement primitives, learning from demonstration, robot safety, nonlinear control

## Abstract

In this paper, we present a novel means of control design for probabilistic movement primitives (ProMPs). Our proposed approach makes use of control barrier functions and control Lyapunov functions defined by a ProMP distribution. Thus, a robot may move along a trajectory within the distribution while guaranteeing that the system state never leaves more than a desired distance from the distribution mean. The control employs feedback linearization to handle nonlinearities in the system dynamics and real-time quadratic programming to ensure a solution exists that satisfies all safety constraints while minimizing control effort. Furthermore, we highlight how the proposed method may allow a designer to emphasize certain safety objectives that are more important than the others. A series of simulations and experiments demonstrate the efficacy of our approach and show it can run in real time.

## 1 Introduction

The idea of proximity between robots and humans performing useful tasks, in a shared work space, inevitably brings up the issue of safety. In fact, safety is a limiting factor for the development of the autonomous robotic partners (Vicentini, 2021). Furthermore, strict safety requirements pose a major challenge for system integrators and robotics applications designers. When humans and robots share a physical work environment, robots must have control laws that make them verifiably safe around humans. In particular, robot systems need to be capable of detecting task variations, and their motion planning and control algorithms must be flexible enough to allow for variation while guaranteeing safety (Kragic et al., 2018).

Robot motion planning is a rich field of study providing myriad approaches to determine robot trajectories, including in the presence of obstacles (Mohanan and Salgoankar, 2018). Many approaches, such as rapidly exploring random trees (LaValle, 1998; LaValle et al., 2001), probabilistic roadmaps (Hsu et al., 1998; Geraerts and Overmars, 2004), and artificial potential fields (Warren, 1989; Vadakkepat et al., 2000) require predefined static maps for peak performance. In addition, robot motion planning algorithms often require expert design of cost functions, potential functions, and random sampling that are outside the expertise of day-to-day users. The learning from demonstration paradigm can address these shortcomings (Argall et al., 2009; Chernova and Thomaz, 2014) by leveraging the inherent expertise of a human teacher.

Movement primitives (MPs) are a popular approach to encode and generalize human demonstrations for training robots. MPs are modeled through a compact representation of the implicitly continuous and high-dimensional trajectories. For example, dynamic movement primitives (DMPs) use demonstrations to learn a model of the control effort necessary to produce a desired trajectory for a stabilized dynamic system (Ijspeert et al., 2013; Dahlin and Karayiannidis, 2019). Capturing the natural variation in human demonstration of a task can help a robot overcome uncertainty and deviation between the training regime and actual task execution (Gomez-Gonzalez et al., 2020). However, DMPs only encapsulate a single trajectory demonstration and thus lack this flexibility.

Probabilistic movement primitives (ProMPs) are a concept in which a *distribution* of trajectories is learned from multiple demonstrations. There are several works that have focused on the construction of ProMP controllers. In (Paraschos et al., 2018a), the design of a stochastic ProMP feedback controller was studied by exploiting the property of the covariance derivatives which can be explicitly computed. A model-free ProMP controller that adapts movement to force-torque input was designed in (Paraschos et al., 2018b). In (Calinon and Lee, 2017), the authors designed a model predictive control-based ProMP controller for a linear discrete time system model.

While they have prominent advantages, ProMP methods still present notable shortcomings. For example, prior ProMP approaches require a linearized model of the system in the controller design. This makes the controller less relevant for nonlinear systems such as robotics and autonomous vehicles. Additionally, while ProMPs themselves are fairly simple to generate, their controllers are difficult to implement, vulnerable to noise, sensitive to design parameters, and are variable to initial conditions. These factors limit the ability of non-experts to employ or tune such controllers. Finally, ProMPs by definition are stochastic and distributions of trajectories defined by Gaussian functions have a large support. Thus, the resulting trajectories can deviate far from the mean of the training set.

In recent years, real-time safety-critical control of dynamic systems has received notable consideration (Wieland and Allgöwer, 2007). One important approach is the use of barrier certificates/functions, which leverage *off-line* iterative optimization algorithms to verify safety for a given dynamical system (Sloth et al., 2012). The notion has been extended to synthesizing safe control laws in real-time using quadratic programming (QP) to find control inputs that satisfy control barrier functions (CBFs) (Ames et al., 2016).

A powerful property of CBFs is that they are easily combined with control Lyapunov functions (CLFs) in the same QP such that the resulting controller guarantees stability while respecting limits and safe regions of the state space (Ames et al., 2019; Cortez et al., 2019; Lopez et al., 2020). Additionally, the QP solved to find a safe, stable control input during run-time can include other optimization terms such as minimizing control effort. Other tasks formulated as cost functions or constraints can be included as well. CBF and CLF based controls have their own downsides, most notably the advanced knowledge necessary to define the barriers and trajectories. Efforts to automate the definition of CBFs and CLFs include mapping temporal logic statements to barriers and trajectories (Srinivasan and Coogan, 2020) and training piece-wise barrier functions for obstacles in the workspace (Saveriano and Lee, 2019).

This work addresses the aforementioned weaknesses of ProMPs and CBFs/CLFs. In our presented approach, the trajectory distribution provided by a ProMP is used to define a CLF and one or more CBFs. Specifically, the ProMP mean is used to define a CLF, and barriers for the CBFs are defined using the standard deviation of the distribution. Thus, the CLF and CBFs are established strictly through human demonstration eschewing the need for advanced control knowledge. The system will roughly track the mean trajectory, with a modicum of freedom to optimize the control effort or other requirements, while guaranteeing that the system never leaves a known neighborhood of the mean.

**FIGURE 1:**
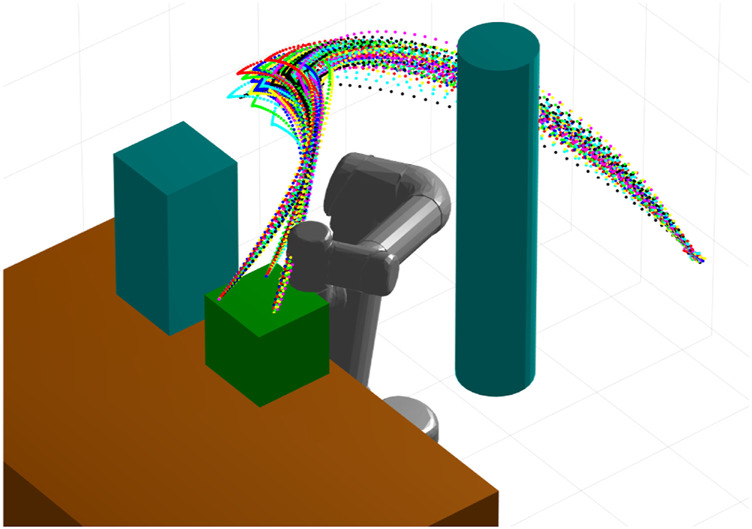
A set of robot trajectories generated by our CLF/CBF-based ProMP demonstration and control method. The controller guarantees that the system never leaves a neighborhood defined by the training set and provides a straightforward way to define trajectories that enforce safety constraints in the presence of obstacles. Copyright 2021 IEEE. Reprinted, with permission, from Davoodi et al., 2021.

Since CLF and CBF controllers are intrinsically based on the nonlinear model of the system, our approach overcomes the inherent linearity of ProMP controllers. We demonstrate the effectiveness and computational efficiency of our approach through simulations and experiments with a two-link and a six-link robot. Examples of generated control trajectories by our method for a Universal Robots UR5 are shown in Figure 1. In summary, the salient contributions of this paper are the following.

• We develop a novel means of automating the design of CLFs and CBFs from the distribution delivered by a ProMP.
• We introduce a new control design for ProMPs by combining CLFs and CBFs.
• We demonstrate the practical applicability of the proposed method through experimental validation on a Universal Robots UR5e.

The remainder of this paper is structured as follows. In Section 2, we review ProMPs, CBFs, CLFs, and robot system dynamics. We detail our approach for a ProMP to define the CLFs and CBFs for optimal control in Section 3. Simulation and experiments are presented in Section 4. Lastly, we conclude with a discussion on future work in Section 5.

## 2 Background

This section presents necessary background information on ProMPs, CBFs, and CLFs.

Notation: Given a matrix *A*, we denote its transpose by *A* ^*⊤*^. Let the identity and zero matrices, with appropriate dimensions, be denoted by *I* and 0, respectively. We denote ⋆ as the symmetric entries of a matrix. For a vector field *f*_*i*_(*x*) and vector of vector fields *F*(*x*) = [*f*_1_(*x*), …, *f*_*n*_(*x*)], let $L_{f_{i}}$ and *L*_*F*_ denote, respectively, the Lie derivative along *f*_*i*_(*x*) and the vector of Lie derivatives in the directions $f_{i}\left(x\right):L_{F}=\left[L_{f_{1}},\ldots ,L_{f_{n}}\right]$ . A continuous function *β*_1_: [0, *a*) → [0, *∞*), for some *a* > 0, is said to belong to class *K* if it is strictly increasing and *β*_1_ (0) = 0. The number of joints for a robot arm is represented by *n*, and a zero-mean i. i.d. Gaussian distribution with mean *m* and (co)variance Σ is denoted N(m,Σ) .

### 2.1 Probabilistic Movement Primitives

ProMPs provide a parametric representation of trajectories which can be executed in multiple ways through the use of a probability distribution. A set of basis functions are used to reduce the model parameters and aid learning over the demonstrated trajectories. The trajectory distribution can be defined and generated in any space that accommodates the system (e.g., joint space or task space) (Paraschos et al., 2018a). In this work we consider joint space trajectories and assume the demonstrations to be normally distributed.

Let $q_{i}\left(t\right)\in R$ be the *i*th state variable. Then $q_{i}\left(k\right)\in R$ is *q*_*i*_(*t*) sampled at time *k*, where *k* ∈ {*t*_1_, … , *t*_*K*_} is a discrete set of sampling times. Within a ProMP, the execution of a trajectory is modeled as the set of robot positions, *ζ*_*i*_ = {*q*_*i*_(*k*)}. Let $w_{i}\in R^{1\times L}$ be a weight matrix with *L* terms. A linear basis function model is then given by

$$x_{i}\left(k\right)=\left[\begin{array}{c} q_{i}\left(k\right)\\ \dot{q_{i}}\left(k\right) \end{array}\right]=\Phi \left(k\right)w_{i}+\xi_{x_{i}},$$

where $\Phi \left(k\right)={\left[\begin{array}{cc} \phi \left(k\right) & \dot{\phi }\left(k\right) \end{array}\right]}^{⊤}\in R^{2\times L}$ is the time-dependent basis function matrix and *L* is the number of basis functions. Gaussian noise is described by $\xi_{x_{i}}∼N\left(0,\Sigma_{x_{i}}\right)$ . Thus, the ProMP trajectory is represented by a Gaussian distribution over the weight vector *w*_*i*_ and the parameter vector $\theta_{i}=\left\{\mu_{w_{i}},\Sigma_{w_{i}}\right\}$ , which simplifies the estimation of the parameters.

We marginalize out *w*_*i*_ to create the trajectory distribution

$$p\left(\zeta_{i},\theta_{i}\right)=\int p\left(\zeta_{i}\;|\;w_{i}\right)p\left(w_{i};\theta_{i}\right)dw_{i}.$$ (1)

Here, the distribution *p* (*ζ*_*i*_, *θ*_*i*_) defines a hierarchical Bayesian model over the trajectories *ζ*_*i*_ (Paraschos et al., 2018a) and $p\left(w_{i}\;|\;\theta_{i}\right)=N\left(w_{i}\;|\;\mu_{w_{i}},\Sigma_{w_{i}}\right)$ . In an MP representation, the parameters of a single primitive must be easy to obtain from demonstrations. The distribution of the state *p* (*x*_*i*_(*k*); *θ*_*i*_) is

$$\;p\left(x_{i}\left(k\right);\theta_{i}\right)=N\left(x_{i}\left(k\right)\;|\;\Phi \left(k\right)\mu_{w_{i}},\Phi \left(k\right)\Sigma_{w_{i}}\Phi {\left(k\right)}^{⊤}+\Sigma_{x_{i}}\right).$$ (2)

The trajectory can be generated from the ProMP distribution using *w*_*i*_, the basis function Φ(*k*), and (2). The basis function is chosen based on the type of robot movement which can be either rhythmic or stroke-based. From (Eq. 2), the mean ${\tilde{\mu }}_{i}\left(k\right)\in R^{2}$ of the ProMP trajectory at *k* is $\Phi \left(k\right)\mu_{w_{i}}$ and the covariance Σ_*i*_(*k*) is $\Phi \left(k\right)\Sigma_{w_{i}}\Phi {\left(k\right)}^{⊤}+\Sigma_{x_{i}}$ .

Multiple demonstrations are needed to learn a distribution over *w*_*i*_. To train a ProMP we use a combination of radial basis and polynomial basis functions. From the demonstrations, the parameters *θ*_*i*_ can be estimated using maximum likelihood estimation (Lazaric and Ghavamzadeh, 2010). However, when there are insufficient demonstrations this may result in unstable estimates of the ProMP parameters. Therefore, similar to (Gomez-Gonzalez et al., 2020), our method uses a regularization to estimate the ProMP distribution. We maximize *θ*_*i*_ for the posterior distribution over the ProMP using expectation maximization,

$$p\left(\theta_{i}\;|\;x_{i}\left(k\right)\right)\propto p\left(\theta_{i}\right)p\left(x_{i}\left(k\right)\;|\;\theta_{i}\right).$$ (3)

In addition, we make use of Normal-Inverse-Wishart as a prior distribution *p* (*θ*_*i*_) to increase stability when training the ProMP parameters (Gomez-Gonzalez et al., 2020).

### 2.2 System Modeling

Consider the following control affine nonlinear system

$$\dot{x}=f\left(x\right)+\tilde{G}\left(x\right)u,$$ (4)

where $x\in R^{n}$ denotes the state, $u\in R^{m}$ is the control input, $\tilde{G}=\left[g_{1},\ldots ,g_{m}\right]$ , and $f:R^{n}\to R^{n}$ and $g_{i}:R^{n}\to R^{n}$ are locally Lipschitz vector fields. It is assumed that the system in (Eq. 4) is controllable.

The model (4) encompasses the dynamic model of robotic manipulators. We consider the following description of robot motion given by the general form by the Euler-Lagrange equations,

$$D\left(q\right)\ddot{q}+H\left(q,\dot{q}\right)=Eu,$$ (5)

where $q\in R^{n}$ are generalized coordinates of the robot, $D\left(q\right)\in R^{n\times n}$ is the inertia matrix, $R^{n\times n}∋H\left(q,\dot{q}\right)=C\left(q,\dot{q}\right)\dot{q}+K\left(q\right)$ is a vector containing the Coriolis and gravity terms, and $E\in R^{n\times p}$ is the actuation matrix that determines the way in which the inputs *u* actuate the system. In this work, we consider the system to be fully actuated (i.e., *p* = *n*), which is typical for robot manipulators. Then, the system description in (Eq. 5) may be converted to an ODE of the form in (Eq. 4) where $x={\left[q,\dot{q}\right]}^{⊤}$ and

$$f\left(x\right)=\left[\begin{array}{c} \dot{q}\\ -D^{-1}\left(q\right)H\left(q,\dot{q}\right) \end{array}\right],\;\tilde{G}\left(x\right)=\left[\begin{array}{c} 0\\ D^{-1}\left(q\right)E \end{array}\right].$$ (6)

### 2.3 Control Barrier and Control Lyapunov Functions

#### 2.3.1 Control Barrier Functions

Let *C* be a set for which we wish to verify that *x*(*t*) ∈ *C*, *∀t*. Then, *C* defines a *safe set*. A smooth function $h\left(x\right):R^{n}\to R$ is defined to encode a constraint on the state *x* of system. The constraint is satisfied if *h*(*x*) ≥ 0 and violated if *h*(*x*) < 0. Concretely, *C* is defined as

$$\begin{array}{cc} & C=\left\{\eta :h\left(\eta \right)\geq 0\right\},\\ & \partial C=\left\{\eta :h\left(\eta \right)=0\right\},\\ & Int\left(C\right)=\left\{\eta :h\left(\eta \right)>0\right\}, \end{array}$$ (7)

where Int(*C*) and *∂C* denote the interior and boundary of *C*, respectively.

Existing approaches to define CBFs include exponential CBFs, reciprocal CBFs, and zeroing CBFs (Ames et al., 2016; Nguyen and Sreenath, 2016). Yet, these methods have trade-offs with respect to ease of definition, boundedness of velocities, speed of convergence, etc. In this work we investigate the use of a reciprocal CBF. This type of CBF has a small value when the states are far from the constraints and it becomes unbounded when the states approach the constraints.

Definition 1 (Ames et al., 2014) Given *C* and *h*, a function B:C→R is a CBF if there exists class *K* functions *α*_1_, *α*_2_, and *α*_3_, and a constant scalar *γ* > 0 such that

$$\begin{array}{cc} & \frac{1}{\alpha_{1}\left(x\right)}\leq B\left(x\right)\leq \frac{1}{\alpha_{2}\left(x\right)},\\ & L_{f}B\left(x\right)+L_{\tilde{G}}B\left(x\right)u-\frac{\gamma }{B\left(x\right)}\leq 0. \end{array}$$ (8)

Remark 1 It is worth noting that based on the definition of the safe set (7), if the initial state of the system is inside the safe set (i.e., *h*(*x*_0_) > 0 when the system’s trajectory gets close to the safety boundary) then the CBF condition forces the systems’ trajectories to go back inside the safe set. This is due to the fact that the derivative of *h*(*x*(*t*)) is negative on the boundary which leads the value of *B*(*x*) (*h*(*x*)) to start decreasing (increasing). Moreover, the constant value *γ* determines how fast the states of the system can reach the safety boundary.

#### 2.3.2 Control Lyapunov Functions

CLFs can be used to model and design dynamical control system inputs to ensure objectives such as stability, convergence to the origin (or other set point), or convergence to a desired trajectory. In order to have a construction similar to CBFs, we consider exponentially stabilizing CLFs (Ames et al., 2014).

Definition 2 In a domain $X\subset R^{n}$ , a continuously differentiable function V:X→R is an exponentially stabilizing CLF (ES-CLF) if *∀x* ∈ *X* there exists positive scalar constants *c*_1_, *c*_2_, *c*_3_ > 0 such that

$$\begin{array}{cc} & c_{1}\|x{\|}^{2}\leq V\left(x\right)\leq c_{2}\|x{\|}^{2},\\ & L_{f}V\left(x\right)+L_{\tilde{G}}V\left(x\right)u+c_{3}V\left(x\right)\leq 0. \end{array}$$ (9)

Having established a CBF to accomplish safety and a CLF to achieve control performance objectives, the two may be unified through a QP. As a result, safe control laws can be computed using the QP to solve the constrained optimization problems at each point in time (Ames et al., 2016).

## 3 Control Development

Our main goal is to design a controller such that the system output tracks a trajectory within the distribution generated by a ProMP. To this end, we first construct a nonlinear inner-loop control law based on the feedback linearization of (Eq. 5). Then, an outer-loop controller established by a CLF-CBF is designed using the distribution parameters ${\tilde{\mu }}_{i}$ and Σ_*i*_. We summarize this process as the following problem objectives.

1. Use the demonstrated trajectories of a robot to train and estimate a ProMP distribution. The ProMP provides the time-varying mean and variance of a trajectory.
2. Develop a feedback linearization controller to obtain a linear and decoupled input-output closed-loop relationship for the error signal.
3. Design a CLF to stabilize the system such that *∀i*, *q*_*i*_ → *μ*_*i*_, where *μ*_*i*_ is the *i*-th element of ${\tilde{\mu }}_{i}$ .
4. Create a CBF to ensure that the error *e*_*i*_ = *q*_*i*_ − *μ*_*i*_ satisfies the safety constraint *∀i*, |*e*_*i*_| < *σ*_*i*_, where *σ*_*i*_ is the (1, 1) element of Σ_*i*_.

**FIGURE 2:**
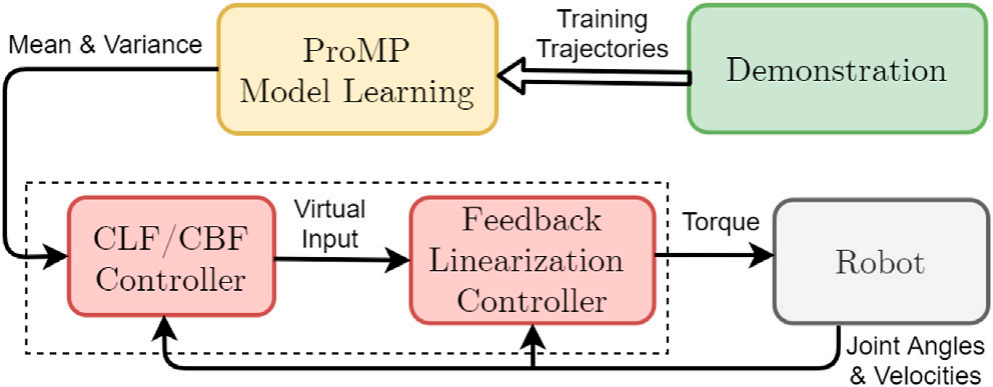
The overall structure of the proposed system.

The general structure of our proposed system is shown in Figure 2.

### 3.1 Feedback Linearization Controller

First, we define the trajectory and error vectors as $\mu ={\left[\mu_{1},\ldots ,\mu_{n}\right]}^{⊤}$ and $e={\left[e_{1},\ldots ,e_{n}\right]}^{⊤}$ , respectively. Using (Eq. 4) and taking the derivative two times along *f*(*x*) and $\tilde{G}\left(x\right)$ , we obtain

$$\begin{array}{ccc} \ddot{e}\left(x\right) & = & L_{f}^{2}e\left(x\right)+\underset{\Gamma }{\underset{︸}{L_{\tilde{G}}L_{f}e\left(x\right)}}u\left(x\right)-\ddot{\mu },\\ \ddot{e}\left(x\right) & = & L_{f}^{2}e\left(x\right)+\Gamma u\left(x\right)-\ddot{\mu }. \end{array}$$ (10)

Next, assume that the decoupling matrix Γ is well-defined and has full rank (Hsu et al., 2015)^1^. This implies that the system in (Eq. 4) is feedback linearizable and we can prescribe the following control law,

$$u\left(x\right)=\Gamma^{-1}\left(-L_{f}^{2}e\left(x\right)+\ddot{\mu }+v\right),$$ (11)

where *v* is an auxiliary feedback control value. This yields the second order linear system from input *v* to output *e*,

$$\ddot{e}=v.$$ (12)

By defining $\eta ={\left[e,\;\dot{e}\right]}^{⊤}$ , (Eq. 12) can be written as a linear time invariant system

$$\dot{\eta }=\left[\begin{array}{cc} 0 & I\\ 0 & 0 \end{array}\right]\eta +\left[\begin{array}{c} 0\\ I \end{array}\right]v.$$ (13)

From there, *n* decoupled systems can be obtained from (Eq. 13),

$${\dot{\eta }}_{i}=F\eta_{i}+Gv_{i},\;\;\;i=1,\ldots ,n,$$ (14)

where $\eta_{i}={\left[e_{i},\;{\dot{e}}_{i}\right]}^{⊤}$ , $F=\left[\begin{array}{cc} 0 & 1\\ 0 & 0 \end{array}\right]$ , and *G* = [0 1]^*⊤*^.

It should be noted that the derivative of the tracking error in (Eq. 14) can be obtained from $\dot{q}$ and $\dot{\mu }$ . Indeed, we can measure $\dot{q}$ and we know *μ* from the ProMP. Since *μ* is a stored trajectory there is no noise in this signal. Therefore, $\dot{\mu }$ can be calculated via backwards difference and it can be made as smooth as necessary (i.e., by creating spline curve from fit points in *μ*). Moreover, the backward difference method is again used for approximating $\ddot{\mu }$ , in (Eq. 10) and (Eq. 11).

To accomplish problem objective 3, it is sufficient to ensure *e*_*i*_ → 0. This is accomplished by designing an appropriate CLF. To satisfy problem objective 4, it is adequate to make |*e*_*i*_| < *σ*_*i*_. This objective is satisfied by defining suitable CBFs. In the following subsections, the CLFs and CBFs are defined for the system in (Eq. 14). Moreover, the *i*th controller for each system in (Eq. 14) is designed by combining the corresponding CLFs and CBFs *via* a QP problem.

### 3.2 Control Lyapunov Function

Consider the following rapidly exponentially stabilizing-CLF (RES-CLF) (Zhao et al., 2014),

$$V_{\epsilon_{i}}\left(\eta_{i}\right)=\eta_{i}^{⊤}\left[\begin{array}{cc} 1/\epsilon_{i} & 0\\ 0 & 1 \end{array}\right]P\left[\begin{array}{cc} 1/\epsilon_{i} & 0\\ 0 & 1 \end{array}\right]\eta_{i},$$ (15)

where *ϵ*_*i*_ is a positive scalar and $P\in R^{2\times 2}$ is a symmetric positive definite matrix that can be obtained by solving the continuous time algebraic Riccati equation

$$F^{⊤}P+PF-PGG^{⊤}P+I=0.$$ (16)

In order to exponentially stabilize the system, we want to find *v*_*i*_ such that

$${\dot{V}}_{\epsilon_{i}}\left(\eta_{i}\right)=L_{F}V_{\epsilon_{i}}\left(\eta_{i}\right)+L_{G}V_{\epsilon_{i}}\left(\eta_{i}\right)v_{i}\leq -\frac{c_{3i}}{\epsilon_{i}}V_{\epsilon_{i}}\left(\eta_{i}\right),$$ (17)

where *c*_3*i*_ is a positive constant value. To guarantee a feasible solution for the QP, the CLF constraint can be relaxed by *δ*_*i*_ > 0 (Ames et al., 2014) resulting in

$$L_{F}V_{\epsilon_{i}}\left(\eta_{i}\right)+L_{G}V_{\epsilon_{i}}\left(\eta_{i}\right)v_{i}+\frac{c_{3i}}{\epsilon_{i}}V_{\epsilon_{i}}\left(\eta_{i}\right)\leq \delta_{i}.$$ (18)

This relaxation parameter will be minimized in the QP cost function. It is worth mentioning that by providing a weighting factor on the relaxation parameter *δ*_*i*_, the QP can prioritize how close the system should track of a specific trajectory while ensuring that safety is always satisfied.

### 3.3 Control Barrier Functions

We propose two safety constraints for each system in (Eq. 14). More specifically, each system should satisfy − *σ*_*i*_ < *e*_*i*_ < *σ*_*i*_. Consequently, we have the following two safety constraints,

$$\begin{array}{cc} h_{i1} & =e_{i}+\sigma_{i},\\ h_{i2} & =-e_{i}+\sigma_{i}. \end{array}$$ (19)

In this work, we assume that the initial conditions satisfy the safety constraints, i.e., the initial tracking errors are in the safe region. From (Eq. 19), it is clear that we have multiple time-varying constraints that should be satisfied simultaneously. Moreover, it is trivial to verify that *L*_*G*_ *e*_*i*_ = 0 and *L*_*F*_ *L*_*G*_ *e*_*i*_ ≠ 0, thus the safety constraint has a relative degree of 2. For relative degree-two constraints, the reciprocal CBF is defined as (Hsu et al., 2015),

$$B_{j}\left(\eta_{i}\right)=-\ln\left(\frac{h_{ij}\left(\eta_{i}\right)}{1+h_{ij}\left(\eta_{i}\right)}\right)+a_{Eij}\frac{b_{Eij}{\dot{h}}_{ij}{\left(\eta_{i}\right)}^{2}}{1+b_{Eij}{\dot{h}}_{ij}{\left(\eta_{i}\right)}^{2}},$$ (20)

where *j* ∈ {1, 2} and *a*_*Eij*_, *b*_*Eij*_ are positive scalars. The following control barrier condition should be satisfied for time varying constraints which leads to time varying CBFs,

$$L_{F}B_{j}\left(\eta_{i}\right)+L_{G}B_{j}\left(\eta_{i}\right)v_{i}+\frac{\partial B_{j}\left(\eta_{i}\right)}{\partial t}-\frac{\gamma_{i}}{B_{j}\left(\eta_{i}\right)}\leq 0.$$ (21)

Remark 2 Note that by choosing small values for *a*_*Eij*_ and *b*_*Eij*_, the system will stop far from the constraint surfaces. On the other hand, by choosing large parameters the system will stop close to the constraints. In some cases, especially in the presence of uncertainties, choosing *a*_*Eij*_ and *b*_*Eij*_ to be too large may cause constraint violations (i.e., no solution exists to the QP problem). As a result, based on the given application, a compromise must be considered for choosing these parameters.

### 3.4 Quadratic Program

As shown in (Eq. 19), two safety constraints need to be satisfied simultaneously for each linearized, decoupled system. Due to this fact, a single controller can be obtained in such a way that guarantees adherence to both constraints (Rauscher et al., 2016). In this subsection, *n* QPs are proposed to unify RES-CLF and CBFs for each system in (Eq. 14) into a single controller. The *n* QPs for *i* ∈ {1, … , *n*} are defined as

$$\begin{array}{cc} & \;\underset{v_{i}={\left[v_{i},\;\delta_{i}\right]}^{⊤}\in R^{2}}{min}\;v_{i}^{⊤}H_{i}v_{i},\\ & subject\;to\\ & L_{F}V_{\epsilon_{i}}\left(\eta_{i}\right)+L_{G}V_{\epsilon_{i}}\left(\eta_{i}\right)v_{i}+\frac{c_{3i}}{\epsilon_{i}}V_{\epsilon_{i}}\left(\eta_{i}\right)\leq \delta_{i}\;\;\;\left(CLF\right)\\ & L_{F}B_{j}\left(\eta_{i}\right)+L_{G}B_{j}\left(\eta_{i}\right)v_{i}+\frac{\partial B_{j}\left(\eta_{i}\right)}{\partial t}\leq \frac{\gamma_{i}}{B_{j}\left(\eta_{i}\right)}\;\;\left(CBFs\right) \end{array}$$ (22)

where $H_{i}=\left[\begin{array}{cc} 1 & 0\\ 0 & p_{sci} \end{array}\right]$ and $p_{sci}\in R_{+}$ is a variable that can be chosen based on the designer’s assessment of weighting the control inputs. Based on the QP problem, if the system states *η*_*i*_ are far away from the boundary of the safe set, then the control objective that is represented by RES-CLF will be satisfied. However, as the states get close to the boundary the control performance will be violated by the CBF.

Remark 3 If feedback linearization is not feasible, or in the case that feedback linearization does not result in independent systems, we should encode the coupling between the joints to train a single ProMP for the multidimensional system. Our proposed approach can be extended to address such cases by defining the safety constraints based on the entire ProMP covariance matrix, not just the diagonal elements. This is an avenue of future exploration.

## 4 Simulations and Experiments

In this section, we demonstrate different aspects and capabilities of our methodology for the ProMP control of a robotic system *via* simulations and experiments. The system models and proposed real-time controller were implemented in a MATLAB 2019a environment. All computations were run on a Dell OptiPlex 7050 machine with an Intel Core i7-7700X CPU and 8 GB of memory.

### 4.1 Case Study 1: Two-Link Robot

We consider a rigid, two-link robot with the dynamic model of (Eq. 5) and the following parameters (Kolhe et al., 2013),

$$\begin{array}{cc} D\left(q\right) & =\left[\begin{array}{cc} m_{1}l_{1}^{2}+m_{2}\left(l_{1}^{2}+l_{2}^{2}+2l_{1}l_{2}\cos\left(q_{2}\right)\right) & ⋆\\ m_{2}\left(l_{2}^{2}+l_{1}l_{2}\cos\left(q_{2}\right)\right) & m_{2}l_{2}^{2} \end{array}\right], \end{array}$$

$$\begin{array}{cc} C\left(q,\dot{q}\right) & =\left[\begin{array}{c} -m_{2}l_{1}l_{2}\sin\left(q_{2}\right){\dot{q}}_{2}\left(2{\dot{q}}_{1}+{\dot{q}}_{2}\right)\\ m_{2}l_{1}l_{2}{\dot{q}}_{1}^{2}\sin\left(q_{2}\right) \end{array}\right],\\ K\left(q\right) & =\left[\begin{array}{c} \left(m_{1}+m_{2}\right)gl_{1}\sin\left(q_{1}\right)+m_{2}gl_{2}\sin\left(q_{1}+q_{2}\right)\\ m_{2}gl_{2}\sin\left(q_{1}+q_{2}\right) \end{array}\right], \end{array}$$

where *m*_1_ and *m*_2_ are the link masses, *l*_1_ and *l*_2_ are the lengths of the links, and *g* is the gravitational acceleration. For the simulations, the values of these variables are selected as *m*_1_ = 1, *m*_2_ = 1, *l*_1_ = 1, *l*_2_ = 1, and *g* = 9.8.

**FIGURE 3:**
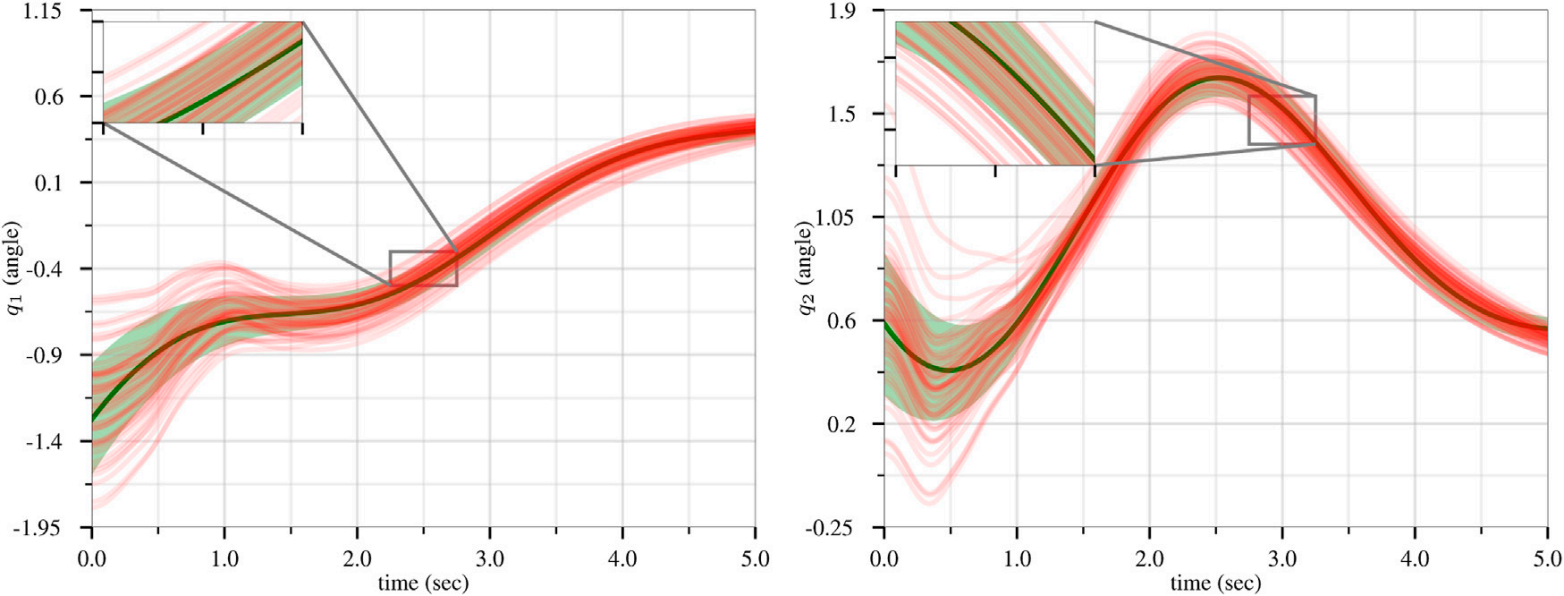
Training of the CLF/CBF-based ProMPs for the first joint (left) and second joint (right). The 50 input trajectories are shown in red, and the ProMP mean joint trajectories (*μ*_*i*_) are shown in dark green. A light-green fill shows *μ*_*i*_ ± *σ*_*i*_. Copyright 2021 IEEE. Reprinted, with permission, from Davoodi et al., 2021.

**FIGURE 4:**
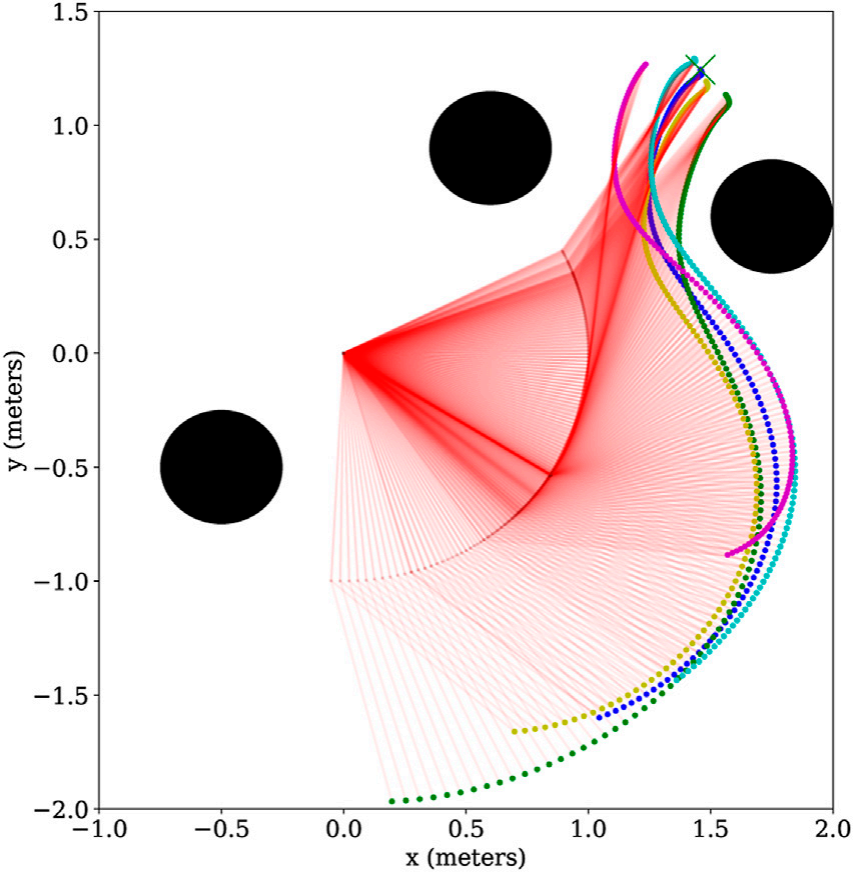
A visualization of the two-link CLF/CBF-based ProMP joint trajectories in the robot workspace. The robot link positions over time are shown in red, while the end-effector trajectory following the mean ProMP joint trajectories is shown in green. The four trajectories of the end effector with joint combinations *μ*_*i*_ ± *σ*_*i*_, *i* ∈{1,2} are illustrated in cyan, blue, yellow and magenta. The black circles correspond to obstacles. Copyright 2021 IEEE. Reprinted, with permission, from Davoodi et al., 2021.

We generated 50 trajectories that achieve a goal position from various starting positions while avoiding three obstacles. Using this dataset, we trained a ProMP with Algorithm 1 from (Gomez-Gonzalez et al., 2020). We used *L* = 2 basis functions consisting of five radial basis parameters. The results of the ProMP training are presented in Figure 3, where the 50 input trajectories are shown in red. The ProMP mean joint trajectories, *μ*_*i*_, are shown in dark green, and in a light-green fill we show *μ*_*i*_ ± *σ*_*i*_. A visualization of the ProMP in the workspace, based on (Sakai et al., 2018), is displayed in Figure 4, where the black circles indicate the location of obstacles. The robot link positions over time are highlighted in red, with different colors representing key end-effector trajectories from the ProMP. The green trajectory is the mean of the ProMP distribution. The other four trajectories result from combinations of *μ*_*i*_ ± *σ*_*i*_, *i* ∈ {1, 2}.

Three sets of simulations were conducted. In each simulation, the CLF parameters were selected as *ϵ*_*i*_ = 0.1 and *c*_3*i*_ = 0.5. In scenario 1, greater priority was given to the CLF than CBF by choosing a high weight, i.e., *p*_*sc*1_ = *p*_*sc*2_ = 200. Moreover, the CBF design parameters were set to *a*_*E*11_ = *a*_*E*12_ = 20.1, *a*_*E*21_ = *a*_*E*22_ = 20, *b*_*E*11_ = *b*_*E*12_ = 1, *b*_*E*21_ = *b*_*E*22_ = 0.9, *γ*_1_ = 10.1, and *γ*_2_ = 9. In scenario 2, *p*_*sci*_ was chosen as *p*_*sc*1_ = *p*_*sc*2_ = 0.02 which implies that less priority was given to the CLF in comparison with the CBF. Moreover, the CBF parameters in this scenario are the same as the first scenario. To show the effects of changing the CBF parameters *a*_*Eij*_, *b*_*Eij*_, and *γ*_*i*_, we consider another scenario. In scenario 3, *a*_*E*11_ = *a*_*E*12_ = 1.1, *a*_*E*21_ = *a*_*E*22_ = 1.1, *b*_*E*11_ = *b*_*E*12_ = 0.4, *b*_*E*21_ = *b*_*E*22_ = 0.5, *γ*_1_ = 1.3, and *γ*_2_ = 1.51, with *p*_*sci*_ = 0.02 as in the second scenario. Consequently, the effects of changing the CBF parameters can be analyzed by comparing the second and third scenarios.

**FIGURE 5:**
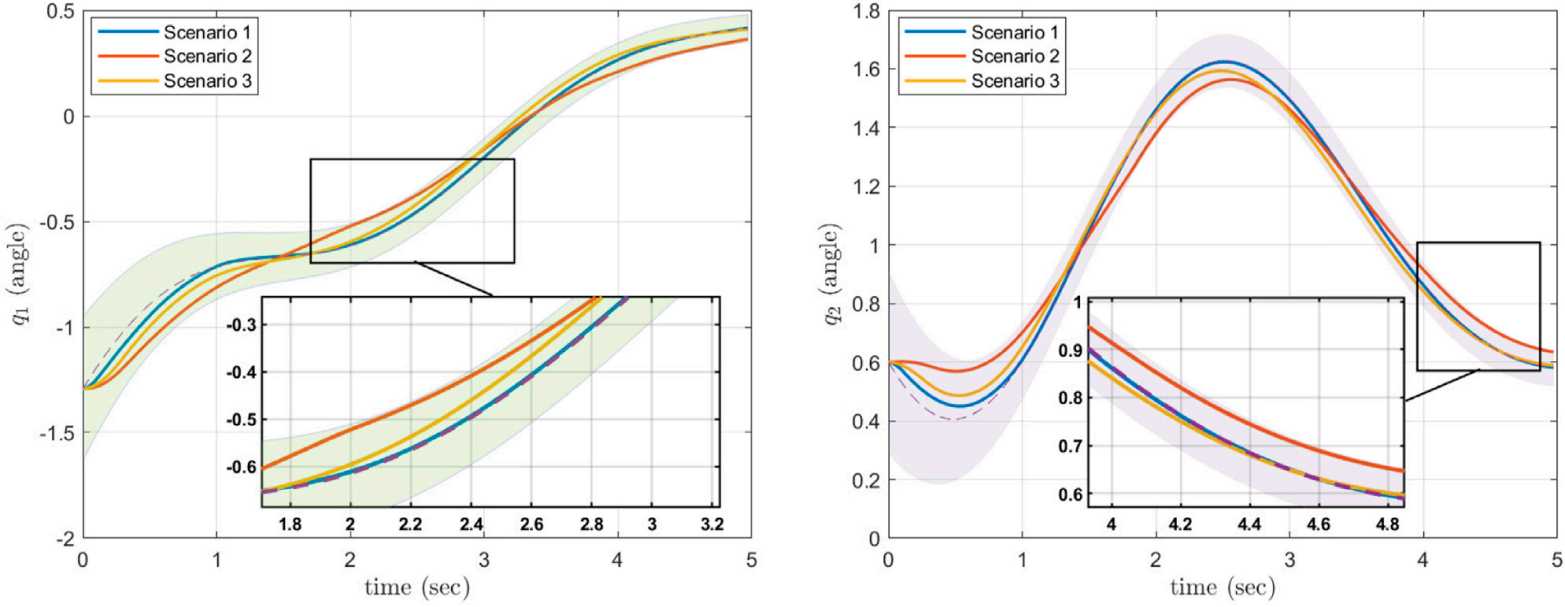
The results of the CLF/CBF-based ProMP controller for the first joint (left) and second joint (right). The safe region of *μ*_*i*_ ± *σ*_*i*_, *i* ∈{1, 2} is shown as a filled “tube.” The control results in different trajectories for distinct values of the weight *p*_*sci*_, however all trajectories remain safe. Copyright 2021 IEEE. Reprinted, with permission, from Davoodi et al., 2021.

The simulation results are exhibited in Figure 5. In scenario 1, by choosing a large value for *p*_*sci*_ (more priority given to the CLF than CBF), the system output remains close to the mean trajectory. However, in scenario 2, by considering a small value for *p*_*sci*_ (more priority given to the CBF than CLF), the system remains safely inside the distribution but does not necessarily stay close to the mean. In scenario 3, it can be seen that by choosing smaller values for *a*_*Eij*_, *b*_*Eij*_, and *γ*_*i*_, the system output will maintain more distance from the constraint surfaces, resulting in remaining closer to the mean trajectory. In short, our proposed method provides a valuable option to the system designer by permitting fine-grained administration of the trajectories while ensuring safety.

The primary computational cost of our controller, with respect to time, comes from the fact that it must solve a set of QPs at every time step. In the evaluation simulations, two QP problems are solved in real time (one for each link). The average required time (*T*_ave_), maximum time (*T*_max_), and the standard deviation (std) for solving the QP problems are *T*_ave_ = {0.0 015*s*, 0.0 011*s*}, *T*_max_ = {0.1 148*s*, 0.0 119*s*}, std = {0.0 053*s*, 0.0 006*s*}. From these results, it is clear that the expected execution time of the QP problems is very small (e.g., in the range of 1 ms). The large maximum times correspond to instances of a single outlier. Hence, the controller is applicable for real-time implementation.

### 4.2 Comparison With Conventional ProMP Control

One specific aspect of the CLF/CBF-based ProMP controller developed in this work is minimizing the control effort in the optimization problem (Eq. 22) at each time step. This leads to a lower control effort in comparison with a traditional ProMP controller. Another set of simulations were conducted to compare our proposed methodology with the results of (Paraschos et al., 2018a; Mathew, 2018), which is representative as one of the primary works in this field. To provide a fair comparison with our method, the ProMP controller is also applied to the feedback linearizable model of the two-link robot.

We conducted three sets of simulations (designated as the fourth, fifth and sixth scenarios). In scenario 4, we implemented the CLF/CBF-based controller with more priority accorded to the CLF rather than the CBF (similar to scenario 1). In scenario 5, we considered the CLF/CBF-based controller with more priority bestowed to the CBF instead of the CLF (similar to scenario 2). The ProMPs in scenarios 4 and 5 were trained using the library in (Gomez-Gonzalez, 2019). However, we were not able to successfully implement the original controller in (Gomez-Gonzalez, 2019). Therefore, in scenario 6, we trained and implemented the traditional ProMP controller presented in (Paraschos et al., 2018a) using the library presented in (Mathew, 2018). While the ProMPs for our CLF/CBF-based ProMP controller and traditional ProMP controller used the same training set, the resulting ProMP mean and covariance are slightly different. For each scenario, the simulations were run for 100 different initial conditions that were randomly selected within the bound [*μ*_1_ ± 0.12, *μ*_2_ ± 0.12], where *μ*_1_ (0) = − 1.292 5, and *μ*_2_ (0) = 0.6.

**FIGURE 6:**
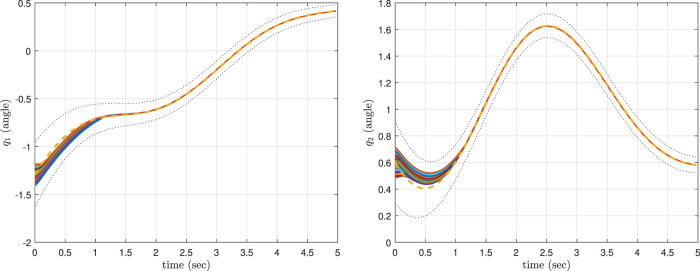
The results of the CLF/CBF-based ProMP controller with more priority given to the CLF than the CBF for the first joint (left) and second joint (right).

**FIGURE 7:**
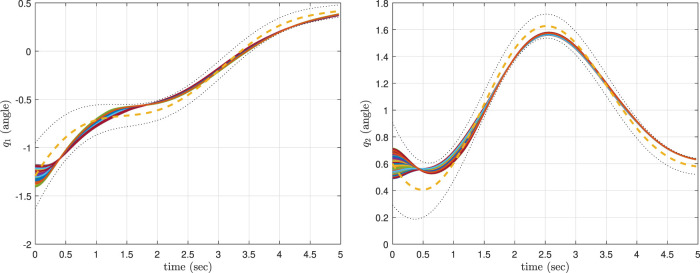
The results of the CLF/CBF-based ProMP controller with more priority given to the CBF than the CLF for the first joint (left) and second joint (right).

**FIGURE 8:**
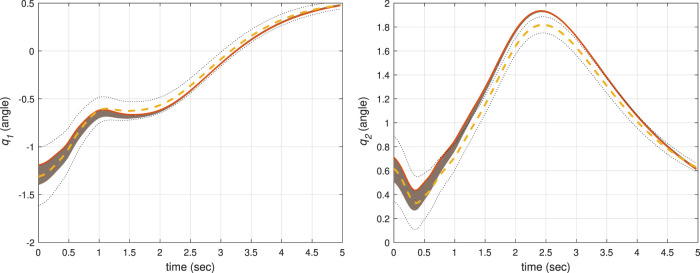
The results of a traditional ProMP controller for the first joint (left) and second joint (right).

The simulation results corresponding to these three scenarios are, respectively, shown in Figures 6–8, where the mean of ProMP is highlighted by a dashed orange line, and the mean ± variance bounds are shown with dotted black lines. These results show that all the controllers are robust against uncertainties in the initial conditions. From Figure 6, it can be concluded that by using the CLF/CBF-based controller with high *p*_*sci*_, the system quickly converges and tracks the mean trajectory. The system deviates from the mean to take a shorter path in Figure 7, but it is clear that by considering small values for *p*_*sci*_ the system remains safely inside the distribution. Based on Figure 8, it can be seen that when using a traditional ProMP controller, the system tracks the mean with an error larger than scenario 4. Moreover, the second joint does not remain inside the distribution during certain periods. We posit that the ProMP controller has some lag, akin to a PID controller with proportional gains that are too low. The feedback and feedforward gains of the ProMP controller are determined as functions of the system parameters and ProMP parameters, and they cannot be tuned to reduce tracking error. The ProMP can be “tuned” through the use of *via* points. To this end, we have added a *via* point as the last element of the mean trajectory to ensure convergence. These results show that the CLF/CBF-based ProMP controller has better tracking performance when compared to a traditional ProMP controller.

**FIGURE 9:**
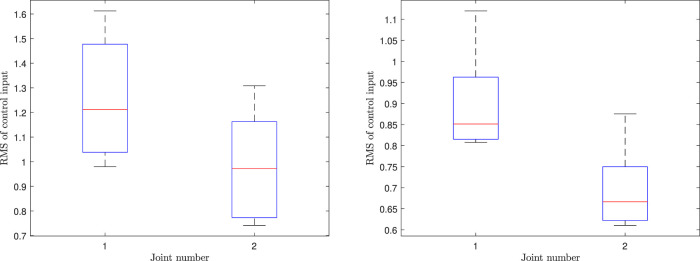
The RMS values of control inputs corresponding to the CLF/CBF-based ProMP controller for the fourth scenario (left) and the fifth scenario (right).

**FIGURE 10:**
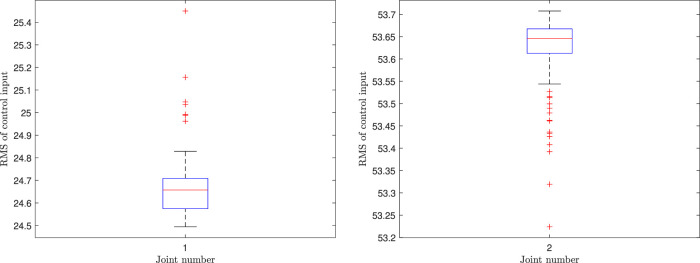
The RMS values of control inputs corresponding to a traditional ProMP controller for the first joint (left) and second joint (right).

Figure 9 and Figure 10 summarize the root mean square (RMS) values of the control variables for scenarios 4, 5, and 6 in the form of a boxplot. In these figures, 50% of all the RMS values are placed in the boxes and the median is shown by a red line that divides the box into two parts. The black bars indicate the maximum and minimum values, and the dashed “whiskers” indicate 25% of the values between the box and max/min. Outliers, if any, are indicated by red crosses. From Figure 9, it can be concluded that scenario 4 has larger control values in comparison to scenario 5, i.e., more control effort is needed to be able to effectively track the ProMP mean. This indicates one strength of leveraging trajectory distributions over a single trajectory; the system is given more freedom to reduce the control effort while maintaining safety. Moreover, in contrast with a traditional ProMP controller our methodology has a remarkably lower control effort as shown in Figure 9 and Figure 10.

### 4.3 Case Study 2: Universal Robots UR5 Six-Link Robot

The equation of motion of the UR5 robot can be written in the form of (Eq. 5), with the following parameters (Spong and Vidyasagar, 2008),

$$D\left(q\right)=\left[\overset{6}{\underset{i=1}{\sum }}m_{i}J_{v_{i}}^{⊤}J_{v_{i}}+J_{w_{i}}^{⊤}R_{i}I_{m_{i}}R_{i}^{⊤}J_{w_{i}}\right],$$ (23)

where $m_{i}\in R$ is the mass of *i*th link, and $J_{v_{i}}\in R^{3\times 6}$ and $J_{w_{i}}\in R^{3\times 6}$ are the linear and angular part of the Jacobian matrix *J*_*i*_, respectively. $R_{i}\in R^{3\times 3}$ is the rotation matrix and $I_{m_{i}}\in R^{3\times 3}$ is the inertia tensor. The elements of $C\left(q,\dot{q}\right)$ are obtained from the inertia matrix as

$$c_{ij}=\overset{6}{\underset{k=1}{\sum }}\frac{1}{2}\left(\frac{\partial m_{ij}}{\partial q_{k}}+\frac{\partial m_{ik}}{\partial q_{j}}-\frac{\partial m_{kj}}{\partial q_{i}}\right){\dot{q}}_{k},$$ (24)

where *m*_*ij*_ are the entries of the inertia matrix. Moreover, the elements of the gravity vector are obtained from

$$K_{i}\left(q\right)=\frac{\partial P}{\partial q_{i}},$$ (25)

where P is the total potential energy of the robot. Additional information on these equations can be found in (Katharina, 2014).

**FIGURE 11:**
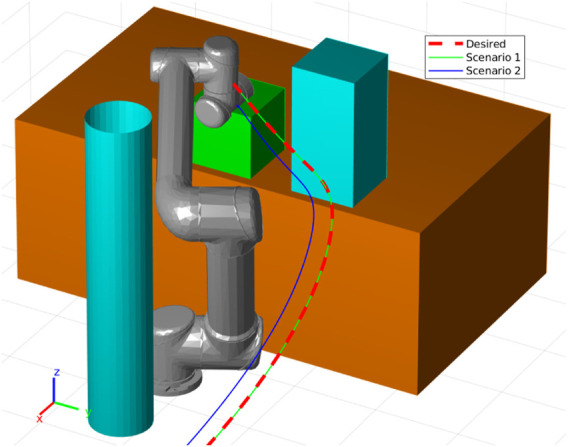
The results of the CLF/CBF-based ProMP controller for a UR5 robot. Copyright 2021 IEEE. Reprinted, with permission, from Davoodi et al., 2021.

We generated 90 joint-space trajectories with defined goals, obstacles, and starting positions. The 90 UR5 trajectories were then used to train a joint-space ProMP using the same parameters as in the two-link robot case study. The following set of CLF and CBF parameters were chosen: *ϵ*_1_ = *ϵ*_2_ = *ϵ*_3_ = *ϵ*_4_ = *ϵ*_5_ = 0.1, *ϵ*_6_ = 0.01, and *c*_3*i*_ = 1.1, *a*_*Eij*_ = 20.1, *b*_*Eij*_ = 1, and *γ*_*i*_ = 10.1, *i* ∈ {1, … , 6}, *j* ∈ {1, 2}. The simulation environment is depicted in Figure 1 and Figure 11. We consider two different scenarios. In scenario 1, *p*_*sci*_ = 200, which gives higher importance to the CLF. For scenario 2, *p*_*sci*_ = 0.001, which implies that the design interest and priority is on the CBF. As is clear from Figure 11, in both scenarios the robot can effectively track the mean of ProMP and simultaneously avoid colliding with environmental obstacles. The running time statistics for solving the QP problems are *T*_ave_ = [0.001 4, 0.001 1, 0.001 0, 0.001 0, 0.001 1, 0.001 0], *T*_max_ = [0.119 3, 0.012 0, 0.012 8, 0.005 8, 0.031 4, 0.002 8], std = [0.005 3, 0.000 5, 0.000 5, 0.000 3, 0.001 4, 0.000 2]. The large maximum values are again due to a single outlier. Thus, the expected operational time is well within the demands of a robotic system. The cause of the outlier occurrences is an avenue of future research to offer improved performance guarantees.

### 4.4 Case Study 3: Universal Robots UR5e Six-Link Robot

To conclude this section, we evaluated our CLF/CBF-based ProMP controller using a physical robot with static obstacles. The robot used was a Universal Robots UR5e six-link manipulator with a Robotiq two-finger gripper. In addition to the robot, our environmental setup included the following three static obstacles: a robotic arm, box, and the table that the robot was mounted on.

We tasked a human teacher with demonstrating a pick-and-place procedure to the robot. In our setup, the robot must move from a starting bin to a goal bin located on the other side of a box. We installed a gravity compensation controller on the robot enabling the teacher to directly affect the robot’s joints via kinesthetic teaching. Using this directed learning from demonstration approach allows us to bypass the correspondence problem (Argall et al., 2009). Additionally, kinesthetic teaching retains parity between the demonstration, learning, and execution space of the trajectories. In all, seven demonstrations of the task were conducted. From these demonstrations, we collected the joint angles, velocities, and the associated time steps. It is worth mentioning that care must be taken to collect data that is roughly Gaussian, or at least unimodal. Methods to handle non-Gaussian or multimodal data is an issue to be addressed in future work.

**FIGURE 12:**
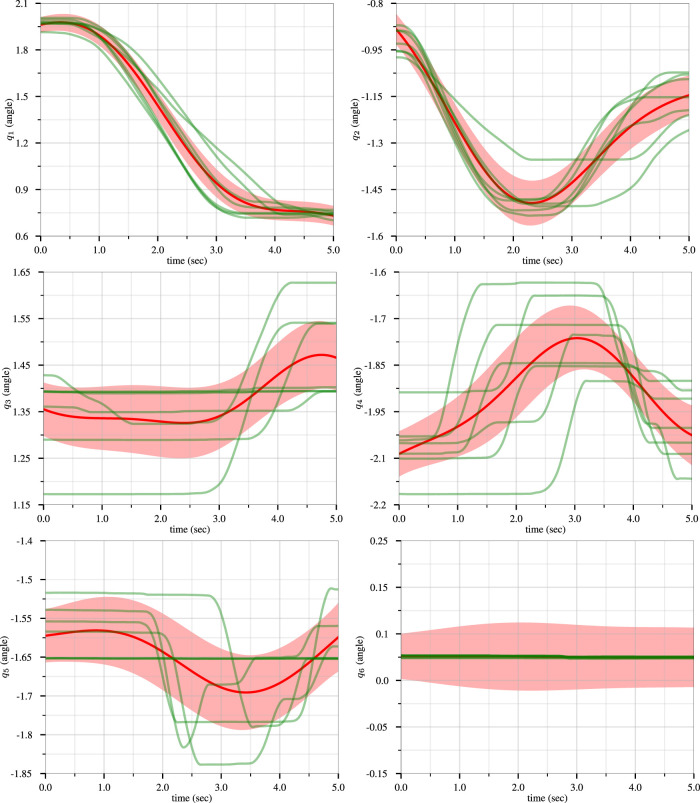
The UR5e robot joint trajectories recorded from the human demonstration of the pick-and-place task are shown in green, while the ProMP mean trajectory and one standard deviation of the ProMP are colored red and light red, respectively.

**FIGURE 13:**
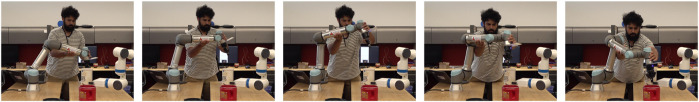
A human teacher demonstrating the pick-and-place task to a UR5e robot.

**FIGURE 14:**
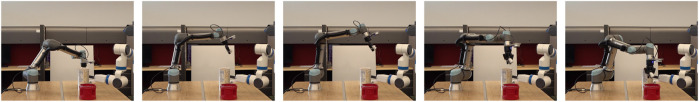
A UR5e robot executing the CLF/CBF-based ProMP controller trajectory.

Using this demonstration data, we trained a joint-space ProMP using the same parameters as described in the previous sections. In Figure 12, we see the demonstration data (in green), the respective ProMP mean (red), and one standard deviation of the ProMP (light red). Note that *q*_6_, which corresponds to the final wrist joint, was not purposefully actuated by the teacher and is largely static during training. We implemented the CLF/CBF-based ProMP controller with *p*_*sci*_ = 0.5. The other parameters were selected to be the same as the previous simulations in Section 4.3. Figure 13 depicts the demonstration operation as the human teacher moves the robot arm during the task. The trajectory from the CLF/CBF-based ProMP controller on the UR5e robot is shown in Figure 14.

## 5 Conclusion and Future Work

In this work we solved a ProMP robot guidance problem using a CLF/CBF-based controller. Our approach stabilizes the robot and guarantees that the system output is always inside the distribution generated by a ProMP. The time-varying nature of the ProMP ensures the robot is guided along the distribution at the desired rate. Moreover, our technique allows for prioritizing between strict tracking of ProMP mean and loose but safe tracking of mean trajectory, which is not possible in the native ProMP control design. It was shown, in Section 4.2, that this can reduce control effort at the risk of getting closer to barriers. Simulation and experimental studies on a two-link and six-link robot confirm the viability of our method for designing the controller.

As part of ongoing work, we are investigating the trade-offs of various different CBFs (e.g., zeroing versus reciprocal), other choices of cost functions, and constraints in the QP. Additionally, we are seeking novel methods that automatically define additional barriers to ensure the safe movement of a co-robot around dynamic obstacles (e.g., humans and other robots). This includes the exploration of an active learning framework whereby a co-robot has the capability to select informative trajectories from the ProMP distribution that can then be used for autonomously retraining itself.

## Data Availability

The data used in this research includes simulation and experimental results. The authors will provide it upon reasonable request.

## Footnotes

1 A control designer would confirm that the system has the same number of inputs as outputs and verify that *e* satisfies a vector relative degree (Kolavennu et al., 2001) condition, typically vector relative degree 2. This implies that the decoupling matrix Γ is well-defined and nonsingular.
